# Piloting a mobile early warning alert and response system for East and Central Darfur, Sudan

**DOI:** 10.1093/inthealth/ihaf122

**Published:** 2025-10-28

**Authors:** Kazuki Shimizu, Muhammad Ali Raja, Muntasir Mohammed Osman, Elfadil Mohammed Mahmoud, Mazza Abasher Alzain, Rizwan Ayub, Marcel Woung, Sara Ahmed, Egmond Samir Evers, Sherein Elnossery, Douaa Fouad Osman Ibragem, Siddeg Khalafalla Ahmed Mustafa, Liesbeth Aelbrecht, Muhammad Fawad Khan, Simon Kaddu Ssentamu, Hala Khudari, Hani Haidar, Shible Sahbani, Haitham Mohamed Ibrahim Awadallah, Boris Igor Pavlin

**Affiliations:** World Health Organization Headquarters, Health Emergencies Programme, Geneva, Switzerland; World Health Organization Regional Office for the Eastern Mediterranean, Health Emergencies Programme, Cairo, Egypt; World Health Organization Country Office in Sudan, Port Sudan, Sudan; World Health Organization Country Office in Sudan, Port Sudan, Sudan; Federal Ministry of Health, Port Sudan, Sudan; Federal Ministry of Health, Port Sudan, Sudan; Federal Ministry of Health, Port Sudan, Sudan; World Health Organization Country Office in Sudan, Port Sudan, Sudan; World Health Organization Headquarters, Health Emergencies Programme, Geneva, Switzerland; World Health Organization Country Office in Sudan, Port Sudan, Sudan; World Health Organization Headquarters, Health Emergencies Programme, Geneva, Switzerland; World Health Organization Regional Office for the Eastern Mediterranean, Health Emergencies Programme, Cairo, Egypt; World Health Organization Regional Office for the Eastern Mediterranean, Health Emergencies Programme, Cairo, Egypt; World Health Organization Country Office in Sudan, Port Sudan, Sudan; World Health Organization Country Office in Sudan, Port Sudan, Sudan; World Health Organization Headquarters, Health Emergencies Programme, Geneva, Switzerland; World Health Organization Regional Office for the Eastern Mediterranean, Health Emergencies Programme, Cairo, Egypt; World Health Organization Country Office in Sudan, Port Sudan, Sudan; World Health Organization Country Office in Sudan, Port Sudan, Sudan; World Health Organization Country Office in Sudan, Port Sudan, Sudan; World Health Organization Country Office in Sudan, Port Sudan, Sudan; World Health Organization Country Office in Sudan, Port Sudan, Sudan; Federal Ministry of Health, Port Sudan, Sudan; World Health Organization Headquarters, Health Emergencies Programme, Geneva, Switzerland; World Health Organization Regional Office for the Eastern Mediterranean, Health Emergencies Programme, Cairo, Egypt; World Health Organization Country Office in Sudan, Port Sudan, Sudan

**Keywords:** epidemiology, health security, humanitarian health, outbreak, surveillance

## Abstract

Conflict in Sudan since April 2023 has disrupted health information systems, particularly in hard-to-reach areas like Darfur. In response to limited surveillance capacity and reports of suspected outbreaks (e.g. measles, cholera, hepatitis E), Sudan’s Federal Ministry of Health, Health Cluster partners and the WHO piloted an emergency surveillance programme using the WHO-developed Early Warning, Alert, and Response System Mobile (EWARS Mobile) in Central and East Darfur states from September 2024 to January 2025. The approach enabled simplified, offline reporting of eight priority diseases and event-based surveillance through partner-supported health facilities. A total of 158 health facilities submitted 752 weekly reports, generating several verified alerts, including suspected measles and acute flaccid paralysis. Despite resource and connectivity challenges, the system provided timely, actionable data for early detection and prompt response, and worked as a beachhead for enhanced partner coordination. The pilot highlighted the value of fit-for-purpose surveillance tools in complex emergencies and demonstrated the feasibility and utility of EWARS Mobile in conflict-affected settings. Based on improved reporting timeliness and partner engagement, the system was approved for expansion to all Darfur states in February 2025.

## Background

The escalation of conflict in Sudan since April 2023 has led to an unprecedented humanitarian health crisis, with >30 million people requiring assistance in 2025.^1^ In the face of disruptions to basic health services, such as vaccination, disease surveillance and public health laboratories, coupled with limited resources and local capacities to detect and respond to outbreaks, disease outbreaks are increasing.^1^ The conflict significantly disrupted the flow of health information from across the country to decision-makers at both state and federal levels, particularly in hard-to-reach regions. Consequently, there has been a lack of reliable data on outbreak-prone diseases and other critical health threats.

Given the extreme vulnerability in the Darfur region amid multiple reports of suspected, presumed and confirmed cases of outbreak-prone diseases such as measles, cholera and hepatitis E, it became essential to establish a simplified, efficient and agile Early Warning, Alert, and Response (EWAR) system for continuous early detection and rapid response to public health hazards affecting emergency-affected populations.^1,2^

Following multiple rounds of consultation involving Sudan’s Federal Ministry of Health (FMoH) and key partners within the national and subnational Health Clusters, the FMoH approved a pilot of a streamlined EWAR surveillance system in June 2024. The pilot was implemented in two of the most inaccessible states: Central Darfur and East Darfur.^1,2^ The WHO-developed Early Warning, Alert, and Response System Mobile (EWARS Mobile; first introduced globally in 2017) software platform—characterised by minimum reporting and manual processing requirements and suitability for low-connectivity settings, was selected for implementation. Data collection was carried out through partner-supported health facilities, which constitute the majority of still-functional facilities in these states (Table 1).

**Table 1.** Timeline of key actions in EWARS Mobile implementation for East and Central Darfur states (February 2024–February 2025)

Date	Key actions
February–April 2024	• The WHO initiated the development of technical guidance documents in coordination with the FMoH and partners.
June 2024	• Agreement reached to pilot EWARS Mobile in Central and East Darfur states. • All technical documents and piloting plans were shared with the FMoH. • The health facility list was reviewed through the Health Cluster.
July 2024	• A hybrid training session was conducted in Port Sudan on 10–11 July, attended by two of the 16 identified health partners. • A meeting with NGO leadership was convened on 31 July to nominate representatives.
August 2024	• A second virtual training session was held on 7 August, with participation from four partners in Central and East Darfur. • A test run was conducted on 27 August with all trained partners, followed by troubleshooting and live reporting. • Continued discussions took place regarding the engagement of additional partners.
September 2024	• Mpox was integrated into the system. • Troubleshooting and live reporting sessions were conducted on 16 September with trained partners. • On 26 September, an agreement was reached with the FMoH on continued WHO headquarters support, including API integration and the initiation of regular FMoH–partners–WHO meetings.
October 2024	• Three partners, including previously trained ones, received additional training. • Regular coordination meetings between the FMoH, partners and the WHO were initiated.
November 2024	• The first pilot phase concluded on 15 November.
December 2024	• The first evaluation meeting was held in a hybrid format. The FMoH provided recommendations focusing on improving timeliness, completeness and expanding engagement with additional partners.
January 2025	• The second pilot phase was initiated.
February 2025	• A follow-up evaluation was conducted in a hybrid format. A decision was made to expand the system to all Darfur states, with minor revisions incorporated in the system.

API: Application Programming Interface; EWARS Mobile: Early Warning, Alert, and Response System Mobile; FMoH: Federal Ministry of Health; NGO: Non-Governmental Organization.

## Approach

### Principles of the EWAR system

EWAR, one of the most critical public health functions in emergencies, refers to systems designed to provide early warnings of acute public health events and to link these alerts to immediate public health responses, thus reducing excess morbidity and mortality caused by epidemic-prone diseases and other public health threats. These threats include severe illnesses with a high case fatality ratio or potential for transmission (e.g. cholera, measles and meningococcal meningitis), emerging and re-emerging communicable diseases including zoonoses, diseases targeted for elimination or eradication (e.g. poliomyelitis) and hazards with the potential for intentional release (e.g. chemical spill). EWAR systems are not designed to capture all diseases of public health importance, particularly those that do not need an immediate response. Additionally, EWAR systems are not intended to track broader indicators relevant in emergencies, such as malnutrition or excess mortality, which require different methodologies.

### Key considerations for EWARS Mobile implementation

There was a debate about whether to expand the existing Sudan EWAR system (Sudan EWARS), which had been implemented in accessible states,^3^ or to introduce EWARS Mobile. While we acknowledge that implementing multiple systems within the same country can be tricky, particularly from the sides of donors and partners, the decision to implement the EWARS Mobile platform was guided by several contextual and operational advantages as follows:

- The mobile application supports data entry via mobile phones in fully offline settings; it also functions offline on computers.
- The system requires minimal information to detect signals, reducing the burden on health workers.
- It integrates community-based event surveillance.
- The platform guides users through the entire process—from detection to alert verification, risk assessment and response—and logs each step.
- Alerts are displayed in real time, without the need for manual data processing and approvals.
- The approach was embraced by key health partners.
- A dedicated development and implementation team maintains the system, which has been refined based on field experiences in similar contexts, including neighbouring Chad^4^ and South Sudan.^5^

### Selection of diseases

**Figure 1.**
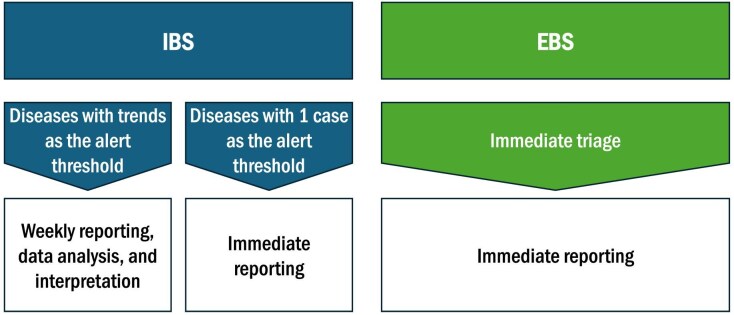
Early warning component of EWAR. EBS: event-based surveillance; EWAR: Early Warning, Alert, and Response; IBS: indicator-based surveillance.

Based on the Early Warning component of EWAR principles^6^ and contextual risk assessments,^1^ and in full consultation with the FMoH, both the indicator-based surveillance (IBS) and event-based surveillance (EBS) components were integrated in the system (Figure 1).

Under IBS, eight priority conditions were selected for monitoring. To simplify reporting while retaining the ability to identify outbreaks, data are not disaggregated by sex or age.

- Immediately notifiable conditions: acute flaccid paralysis (AFP), suspected measles and suspected cholera; and any unusual public health events. Suspected mpox was added to the list in September 2024.
- Weekly reported conditions: suspected meningitis, bloody diarrhoea, acute jaundice syndrome (AJS), severe acute respiratory infection, suspected malaria and confirmed malaria; and total consultations for proportionate morbidity calculation.

In addition, the EBS component has been integrated to enable immediate triage and reporting of any unusual public health events or potential health risks, including clusters of unexplained deaths, chemical spills and animal die-offs.

### Case definition and alert thresholds

To facilitate alignment and future integration into Sudan’s postconflict health information system, case definitions used in EWARS Mobile closely follow those applied in accessible states,^3^ with minor simplifications to enhance usability among partners possessing minimal epidemiological training.

For each disease, alert thresholds have been established, using either fixed numerical values or average-based calculations (e.g. twice the average of the previous 3 wk). These thresholds were guided by previous experience with Sudan EWARS^3^ and similar systems elsewhere, and have been further adjusted based on insights gained since implementation. In the case of the immediately notifiable conditions, the threshold is a single instance. Once the threshold is met, EWARS Mobile initiates an alert management protocol, including verification, risk assessment and risk characterisation.

### Reporting units and locations

EWARS reporting units include primary, secondary and tertiary health facilities operated by partners. Most healthcare facilities operate as primary healthcare centres, and the WHO, as the Health Cluster lead agency, coordinates their operations. Only outpatient services, including emergency departments, are involved in reporting. Not all partners in Central and East Darfur have been trained, and some health facilities supported by trained partners could not be included in reporting due to remoteness or lack of connectivity. Health facilities submit weekly reportable diseases/events, shortly after each epidemiological week, or immediate alerts at any time, through the application. Designated health workers, or data clerks in larger facilities, are responsible for submitting tallies of cases.

## Implementation

The FMoH, partners in the Health Cluster and the WHO jointly implemented the EWARS Mobile pilot in Central and East Darfur. Preparatory activities included a hybrid 2-d training workshop in Port Sudan in July 2024, attended by senior leadership from the FMoH (including the Federal Minister of Health) and the WHO (the WHO Representative in Sudan). The system was implemented entirely using existing resources (e.g. reporters’ phones and in-kind support), as no funding was available for typical costs such as hardware purchases, airtime or reporting incentives.

Following the initial session, additional training was conducted remotely. During the pilot phase, a total of five training sessions with two troubleshooting sessions were delivered to partner organisations (Table 1).

### Data procedure

At the facility level, cases meeting the case definitions are first recorded on tally sheets and subsequently reported either directly into the system or to their organisation’s monitoring or surveillance officer, who enters multiple reports on behalf of their facilities. Surveillance data are compiled and shared with partners through a weekly bulletin. In addition, from late October 2024, the FMoH, the WHO and partner organisations held fortnightly meetings to review the data, discuss emerging trends and guide evidence-based decision-making and response actions.

Unusual events reported through the system are jointly monitored by the FMoH and the WHO. Upon identification of such events, and based on the risk assessment outcomes, rapid actions are taken such as alerting nearby facilities or vaccination teams.

### Surveillance outcomes

From 31 August 2024 to 31 January 2025, a total of 752 weekly reports were submitted from 158 health facilities. As of 15 November 2024, seven alerts were raised: AFP, two suspected measles, AJS and three confirmed malarias. In January 2025, one confirmed outbreak of suspected measles in Central Darfur had been recorded, with pending investigations for AFP in East Darfur, suspected measles in Central Darfur, suspected measles in Central Darfur and AJS in East Darfur.

### Surveillance quality

The quality of the surveillance system was assessed by epidemiological attributes, with a primary focus on completeness, timeliness and representativeness, based on the standardised frameworks developed by the WHO^7^ and the United States Centers for Disease Control and Prevention.^8^ For completeness, 16 out of 19 (84%) known partners reported. For timeliness, the proportion of on-time reports reached a maximum of 33% in early January 2025. For representativeness, all localities in East Darfur and eight out of nine localities in Central Darfur submitted at least one weekly report.

## Discussion

Despite significant challenges, including limited human resources, constrained laboratory capacity and operational difficulties associated with working in remote and conflict-affected settings, EWARS Mobile was successfully piloted in Central and East Darfur at a minimal operational cost (except for staff time: two epidemiologists from the WHO headquarters dedicated 100% of their time for 1–2 mo to strategy planning and 25–50% of their time to support the implementation and sustain the system) and could identify altered possible outbreaks.

Similar to the EWAR system in Yemen,^9^ there was a particular challenge in timeliness, and it was difficult to determine the exact extent to which late reporting was influenced by factors such as weekend clinic closures, limited connectivity or delayed access to mobile networks. Initially, partners with delayed reporting were followed up via E-mail, and later through a WhatsApp (Meta, Inc., Menlo Park, United States) group that included all partners. However, this approach did not significantly improve reporting timeliness. As a result, bilateral communication channels were established with individual partners via WhatsApp. This facilitated more effective follow-up on delayed reports and enabled timely alert verification when necessary. Encouraged by improved completeness and timeliness, and increased partner engagement in January 2025, the FMoH approved the expansion of the system to all five Darfur states in February 2025, where it currently operates across 572 facilities.

The pilot faced several limitations. An absence of baseline surveillance data initially made it difficult to define and calibrate appropriate alert thresholds. Based on lessons learned from Syria on the complementary EWAR system,^10,11^ we placed importance on the previously established local context, while introducing only minor changes. Connectivity issues challenged training and timely reporting. Geographical coverage might have been skewed due to the prioritisation of facilities supported by active partners on a first-come, first-served basis. Therefore, like other early warning systems in the region,^12^ the pilot did not fully encompass all affected populations, and some localised outbreaks in these states might have been undetected. Additional challenges included limited partner presence in certain areas, the absence of an integrated outbreak response strategy, logistical barriers to sample collection and laboratory confirmation, and initially low reporting rates, some of which were also observed during the implementation phase in South Sudan.^5^ Many of these issues were collectively addressed among stakeholders. Future improvements should focus on enhancing ground-level response capacity, including the monitoring, evaluation and training of frontline health workers.

### Conclusion

In resource-constrained and emergency-affected settings, the implementation of surveillance systems must be grounded in a realistic assessment of available resources and logistical feasibility. The EWARS Mobile pilot for Darfur demonstrated its potential to provide actionable, real-time operational data supporting rapid public health responses, thereby reducing morbidity and mortality, in even the most challenging settings. Despite starting with minimal data infrastructure, the system enabled meaningful surveillance and fostered stronger collaboration among partners through the subnational Darfur Health Cluster.

Moving forward, a fit-for-purpose approach, tailored to local capacities and contextual realities, will be essential for scaling and sustaining EWAR systems in Sudan and similar settings.

## Data Availability

The datasets used and/or analysed during the preparation of this manuscript are available from the corresponding author upon reasonable request, subject to consultation with and approval from the FMoH.
